# A Meta-Analysis on Prophylactic Donor Heart Tricuspid Annuloplasty in Orthotopic Heart Transplantation: High Hopes from a Small Intervention

**DOI:** 10.3390/healthcare9030306

**Published:** 2021-03-10

**Authors:** Alberto Emanuel Bacusca, Andrei Tarus, Alexandru Burlacu, Mihail Enache, Grigore Tinica

**Affiliations:** 1 Department of Cardiovascular Surgery, Cardiovascular Diseases Institute, 700503 Iasi, Romania; alberto-bacusca@email.umfiasi.ro (A.E.B.); andrei.tarus@umfiasi.ro (A.T.); mihail.enache@umfiasi.ro (M.E.); grigore.tinica@umfiasi.ro (G.T.); 2 Faculty of Medicine, University of Medicine and Pharmacy “Grigore T Popa”, 700115 Iasi, Romania; 3 Department of Interventional Cardiology, Cardiovascular Diseases Institute, 700503 Iasi, Romania

**Keywords:** heart transplant, tricuspid annuloplasty, tricuspid regurgitation, prophylactic, meta-analysis

## Abstract

(1) Background: Tricuspid regurgitation (TR) is the most frequent valvulopathy in heart transplant recipients (HTX). We aimed to assess the influence of prophylactic donor heart tricuspid annuloplasty (TA) in orthotopic HTX (HTX-A), comparing the outcomes with those of HTX patients. (2) Methods: Electronic databases of PubMed, EMBASE, and SCOPUS were searched. The endpoints were as follows: the overall rate of postprocedural TR (immediate, one week, six months, and one year after the procedure), postoperative complications (permanent pacemaker implantation rate, bleeding), redo surgery for TR, and mortality. (3) Results: This meta-analysis included seven studies. Immediate postprocedural, one-week, six-month and one-year tricuspid insufficiency rates were significantly lower in the HTX-A group. There was no difference in permanent pacemaker implantation rate between the groups. The incidence of postoperative bleeding was similar in both arms. The rate of redo surgery for severe TR was reported only by two authors. In both publications, the total number of events was higher in the HTX cohort, meanwhile pooled effect analysis showed no difference among the intervention and control groups. Mortality at one year was similar in both arms. (4) Conclusion: Our study showed that donor heart TA reduces TR incidence in the first year after orthotopic heart transplantation without increasing the surgical complexity. This is a potentially important issue, given the demand for heart transplants and the need to optimize outcomes when this resource is scarce.

## 1. Introduction

Tricuspid regurgitation (TR) is the most frequent valvulopathy in heart transplant recipients (HTX), with a reported incidence ranging between 19% to 84% [1,2]. The tricuspid valve (TV) integrity manifests a significant impact on the long-term clinical progress and survival of orthotopic HTX. Although most of the patients present a small degree of tricuspid insufficiency, moderate or greater grades were associated with significantly worse survival and higher post-transplant complications [3]. TR etiology is multifactorial, with several viable hypotheses still debatable: biatrial transplantation technique, allograft dysfunction or rejection, donor-recipient size mismatch, or structural damage during endomyocardial biopsy [4,5,6,7,8].

Postoperative moderate or severe TR negatively affects the overall survival rates after HTX [9]. Despite the fact there is a reported improvement of the degree of tricuspid regurgitation six months after the transplantation, the nature of this valvulopathy is progressive. Studies with more extended follow-up periods reported an increase in severe TR incidence from 7.8% at five years to 14.2% at ten years [10].

The most frequently reported indication for heart surgery after HTX was the atrioventricular valve reconstructions or replacement. 62.5% of these cases were related to the tricuspid valve [11]. Surgical repair or replacement is required when right heart failure becomes refractory to conservative medical treatment [10,12]. The mean duration from transplantation to severe TR diagnosis is reported to be 43 +/- 6.38 months [10]. The cardiac mechanics portending right ventricular failure can be accurately predicted using either right cardiac catheterization or by noninvasive methods computational modeling of hemodynamic and cardiac mechanics using lumped-parameter and biventricular finite element analysis [13,14].

To improve the TV function and avoid the risks associated with redo heart surgery, prophylactic tricuspid annuloplasty (TA) on the donor’s heart was proposed as a simple solution to a problem that triggered an increasing concern. Already an established and widely performed surgery, primarily in functional TR treatment, TA accomplished either by DeVega’s technique or by a ring is associated with excellent long-term results [15,16]. TA was envisioned to enhance posttransplant hemodynamics and prevent late moderate/severe TR. Moreover, the importance of TV repair was emphasized not only in heart transplanted patients but also in those receiving left ventricular assist devices either as a bridge therapy or as destination therapy, in which concomitant TV repair may reduce postoperative right ventricular failure [17].

Although a significant reduction in TR after this procedure was reported by most of the authors, actual data are controversial, and opinions regarding its impact on overall survival are heterogeneous. To date, there is no consensus on the concomitant management of the TV during heart transplant [18].

The purpose of this study is to assess the influence of prophylactic donor heart tricuspid annuloplasty (in terms of postoperative complications, effects on hemodynamic parameters, short- and long-term tricuspid regurgitation, and mortality) in orthotopic heart transplant recipients.

## 2. Materials and Methods

The preferred reporting items for systematic reviews and meta-analysis (PRISMA) checklist was applied in each step of the meta-analysis conduction (Supplementary Table S1).

### 2.1. Search and Eligibility

We performed an extensive search for studies comparing heart transplantation with and without prophylactic tricuspid annuloplasty in three electronic databases: PubMed, EMBASE, and SCOPUS from inception to 20th December 2020. We used the following interrogation terms: “heart transplantation,” “tricuspid regurgitation,” “tricuspid valvuloplasty,” “de Vega.” Two independent authors (A.E.B. and A.T.) checked titles and abstracts for eligibility. Fulltext was retrieved for selected papers and verified for fulfilling the following inclusion criteria: (1) study design—randomized control trials, observational studies, propensity score match studies; (2) population—patients with orthotopic heart transplantation; (3) intervention—donor heart tricuspid annuloplasty; (4) comparators—heart transplanted patients without prophylactic tricuspid annuloplasty; (5) outcomes—reported at least post-transplantation tricuspid regurgitation. Both authors scanned the references in relevant articles. The third reviewer (G.T.) mediated the situations when consensus regarding a manuscript’s inclusion was not achieved.

### 2.2. Intraoperative Timing and Outcomes

We compared intraoperative timing between two cohorts (ischemic time, cardio-pulmonary bypass time, and cross-clamp time). The endpoints were as follows: the overall rate of postprocedural TR (immediate, one week, six months, and one year after the procedure), postoperative complications (permanent pacemaker implantation rate, bleeding), redo surgery for TR, and mortality.

### 2.3. Data Collection and Synthesis

The same reviewers extracted data only from retrieved published manuscripts and registered them in standard tables. When the ratio of events and not raw data were available, we calculated the event number from the described ratio and total cohort.

Review Manager (RevMan) Version 5.3 (Nordic Cochrane Centre, The Cochrane Collaboration, 2012, Copenhagen, Denmark) software was used to generate the pooled effect size with odds ratio (OR) and 95% confidence intervals (CI) by Mantel–Haenszel method and random effect model for dichotomous data. A *p*-value of less than 0.05 was considered significant. Conversion to mean and standard deviation (SD), when median and IQR were available, was performed following the methods published by Luo et al. and Wan et al. [10,11]. The pooled sample mean and pooled standard deviation for selected studies were calculated according to the Cochrane Handbook’s recommendation for Systematic Reviews. We used MedCalc Statistical Software version 14.8.1 (MedCalc Software bvba, Ostend, Belgium; http://www.medcalc.org (accessed on 20 December 2020); 2014) for comparative statistics. Chi-squared and *t*-Student’s tests were used to compare dichotomous and continuous data.

### 2.4. Studies Quality Assessment

The risk of publication bias was assessed with the Newcastle–Ottawa quality assessment scale (NOS) for cohort studies and the Cochrane risk of bias tool for randomized controlled trials.

## 3. Results

### 3.1. Literature Search and Study Selection

**Figure 1:**
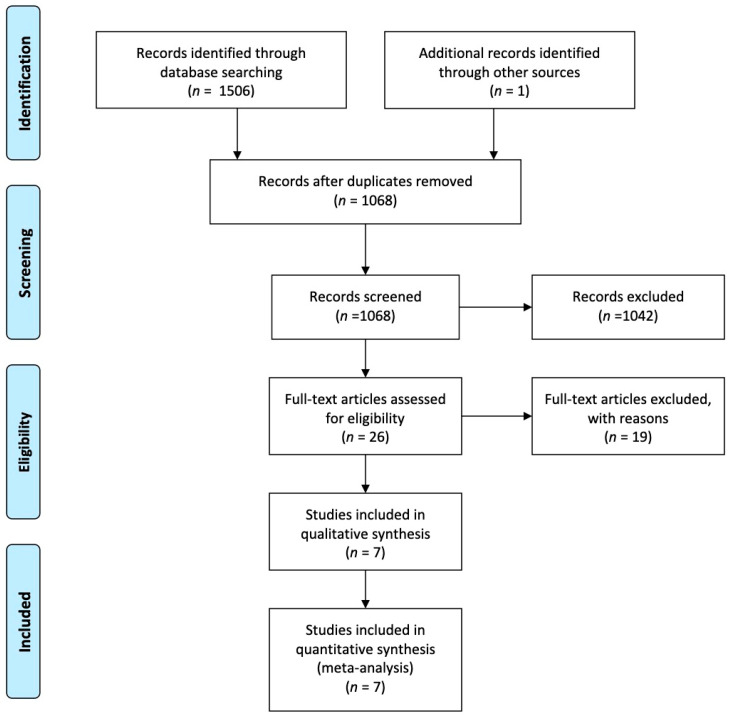
Preferred reporting items for systematic reviews and meta-analysis (PRISMA) flow diagram for study selection.

The digital search identified a total of 1506 titles. After duplicates removal, a total of 1068 references were screened by title and abstract. There were 26 articles selected for full-text analysis (Figure 1).

Seven full-text articles that compared the incidence of moderate or severe tricuspid regurgitation, postoperative complications, and late mortality in heart transplant patients with donor tricuspid annuloplasty with cohorts with no prophylactic tricuspid valve repair during OHT were retrieved [2,19,20,21,22,23,24]. Two of the studies had the same cohort of patients and reported the same outcomes at different periods [22,23]. Two other studies have been conducted by the same authors in the same center [2,20]. The criteria for patient selection and the reported outcomes were the same. We have considered the data presented in the most recent study that also included the more representative cohorts of patients.

### 3.2. Study Characteristics and Risk of Bias

The characteristics of the selected studies are presented in Table 1. All studies were appreciated to have a good quality design (Supplementary Table S2).

### 3.3. Patient and Periprocedural Characteristics

The final analysis included 730 patients, of which 359 heart transplant recipients with prophylactic donor tricuspid annuloplasty (HTX-A) and 371 patients without tricuspid valve repair (HTX group). Both bicaval and biatrial heart transplantation techniques were taken into account. De Vega and Ring tricuspid valve annuloplasty procedures were analyzed.

Baseline characteristics and periprocedural data distinguishing each group are summarized in Table 2. Patients in both groups predominantly male and had similar ages.

Ischemic etiology of the end-stage heart failure was more frequent in the HTX group (88.63% vs. 67.57%, *p* = 0.0001). There was no difference in preoperative renal status, mechanical circulatory support, or inotropic drug use. The pulmonary capillary wedge pressure was higher in the HTX-A group (19.70 ± 9.13 vs. 17.15 ± 8.54, *p* = 0.0047), but pulmonary vascular resistance was similar.

### 3.4. Intraoperative Times

Intraoperative data analysis revealed longer cardiopulmonary bypass time (173.32 ± 27.75 vs. 154.14 ± 25.88, *p* < 0.0001) and ischemic time (181.75 ± 40.82 vs. 165.31 ± 41.72, *p* < 0.0001) in the HTX group, but no difference in the aortic cross-clamp time.

### 3.5. Outcomes

#### 3.5.1. Tricuspid Regurgitation

**Figure 2:**
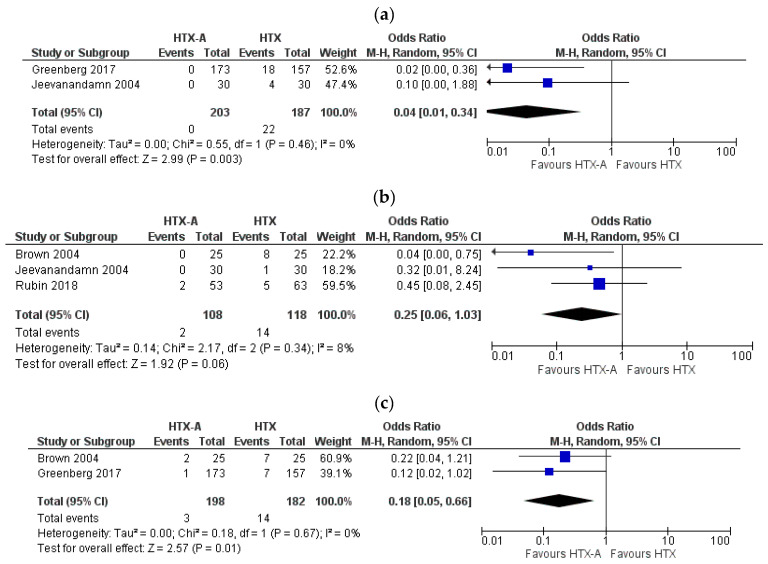

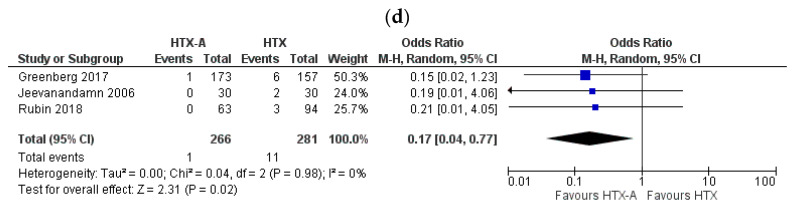
Forest plot depicting post-transplantation TR: (**a**) immediate; (**b**) after 1 week; (**c**) after 6 months; (**d**) after 1 year.

Forest plots for postoperative TR in different periods are shown in Figure 2a–d. Immediate postprocedural, one week, six months and one year tricuspid insufficiency rate was significantly lower in HTX-A group (HTX-A vs. HTX: OR: 0.04, 95% CI, 0.01 to 0.34, I^2^ = 0%); (HTX-A vs. HTX: OR: 0.25, 95% CI, 0.06 to 1.03, I^2^ = 8%); (HTX-A vs. HTX: OR: 0.18, 95% CI, 0.05 to 0.66, I^2^ = 0%); (HTX-A vs. HTX: OR: 0.17, 95% CI, 0.04 to 0.77, I^2^ = 0%).

#### 3.5.2. Periprocedural Complications

**Figure 3:**
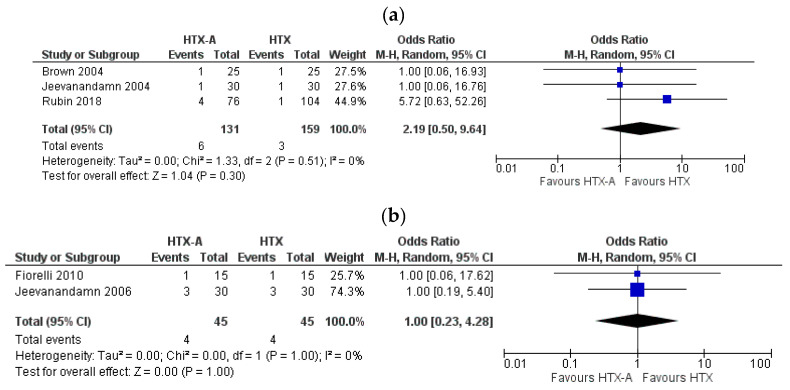
Forest plot depicting periprocedural complications: (**a**) permanent pacemaker implantation rate; (**b**) postoperative severe bleeding rate.

There were no difference in permanent pacemaker implantation rate between the goups (HTX-A vs. HTX: OR: 2.19, 95% CI, 0.50 to 9.64, I^2^ = 0%) (Figure 3a). Incidence of postoperative bleeding was similar in both arms (HTX-A vs. HTX: OR: 1.00, 95% CI, 0.23 to 4.28, I^2^ = 0%) (Figure 3b).

#### 3.5.3. Reoperation and Survival

**Figure 4:**
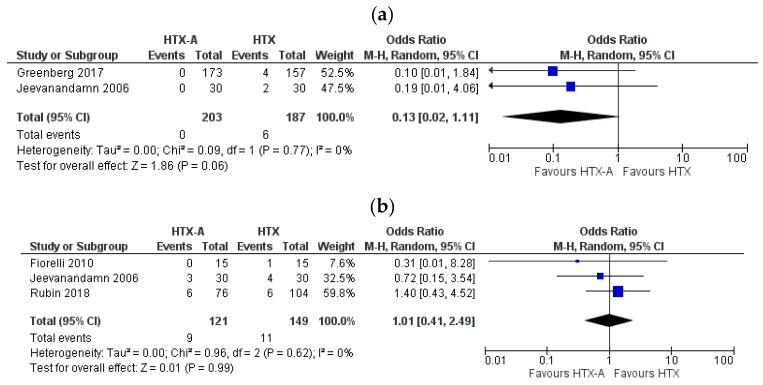
Forest plot depicting: (**a**) reoperation rate on tricuspid valve after transplantation; (**b**) 1-year survival.

The rate of redo surgery for severe TR was reported only by two authors. In both publications, the total number of events was higher in the HTX cohort, meanwhile pooled effect analysis showed no difference among the intervention and control groups (HTX-A vs. HTX: OR: 0.13, 95% CI, 0.02 to 1.11, I^2^ = 0%) (Figure 4a). Mortality at 1 year was similar in both arms (HTX-A vs. HTX: OR: 1.01, 95% CI, 0.41 to 2.49, I^2^ = 0%) (Figure 4b).

## 4. Discussion

Our meta-analysis shows that donor heart tricuspid annuloplasty reduces tricuspid regurgitation incidence in the first year after orthotopic heart transplantation without increasing the surgical complexity. No significant benefit or harm was revealed on long-term mortality. Performed in high-experienced centers, prophylactic donor tricuspid annuloplasty could be routinely considered during orthotopic heart transplantation as it tends to incline the balance to a more favorable evolution.

Tricuspid regurgitation is a common problem after heart transplantation. There are two main types of tricuspid insufficiency. Type I dysfunction is more common and occurs earlier, with a reported average time from the procedure to the onset of severe TR of 13 months [25]. In this scenario, the regurgitation is due to the alteration in the TV geometry and right atrium, followed by annular/ventricular dilation. The tricuspid valve leaflet motion is normal. Evolution under medical therapy is usually mild but may become severe and require surgical correction [25,26].

Type II dysfunction has a reported average time to onset of severe TR of 28 months and is characterized by an excessive leaflet motion mostly due to chordal disruption after right ventricular endomyocardial biopsy [25]. Mild to moderate TR may be well-tolerated, but recurrent injury or spontaneous rupture of the chordae tendineae could also lead to severe symptomatic TR that may require surgical repair [27,28].

The etiology of the disease is multifactorial. In a multivariate analysis, the standard biatrial transplantation technique is considered the most independent predictor for early and late TR in heart transplant recipients [5]. Due to a higher distortion and dilatation of the tricuspid annulus, biatrial transplantation can lead to a more frequent and severe type I tricuspid regurgitation in all time scales following transplantation. After a one-year follow-up, the patients who underwent transplantation by the biatrial technique showed higher right-sided pressures and thus added another risk factor in developing the TR [5].

Despite these findings, some authors disagree with this hypothesis. Kim and colleagues found that the occurrence of TR was not related to the anastomosis technique [29], and Kalra et al. revealed in an echocardiographic study comparing bi-caval versus atrial anastomosis technique, no effect of the technique on tricuspid regurgitation [30]. Another study identified that the strongest predictor of moderate to severe TR would rather be the presence of intraoperative RV dysfunction [3]. Other risk factors associated with the development of type I TR are the donor age, the preoperative pulmonary hemodynamics, pre-transplant dilated cardiomyopathy weight mismatch, and more than two cellular rejection episodes [5,9,29].

The development of long-term significant type II TR after transplantation was correlated with the number of endomyocardial biopsies performed (EMB) [5]. (*A significant correlation between the occurrence of tricuspid valve injury and EMB number performed per patient was observed* [12].) Percutaneous transvenous EMB remains the most suitable method for the early identification of histopathologic alterations; thus, the gold standard in the diagnose of cardiac rejection [31]. The reported TR caused by iatrogenic injury during EMB was 6–32% of cases [3,32,33], and almost half of all myocardial fragments recovered from patients with significant TR revealing the presence of chordae tendineae [12]. The risk factors of developing tricuspid injury are EMB technique, bioptome type, method of bioptome guidance, and access route and team experience [12,32,34]. Noninvasive methods sought to replace the EMB yet did not prove able to overcome histological analysis’s advantages [35,36]. Gallium-67 scintigraphy used as a screening method has resulted in favorable outcomes, with an approximately 10-fold reduction of EMB per patient [37]. Although TV annuloplasty is performed to maintain the annulus’s standard size, minor structural damage caused by EMB could also be attenuated due to the annulus reduction [23].

The impact of TR on transplantation outcomes is unquestionable. Anderson and colleagues report a 38% operative mortality in patients with mild or greater severity TR versus 7% in patients with no or trace TR. In the absence of RV dysfunction, one-year survival rates were 92% for those with no or trace TR vs. 57% with mild or greater severity TR. A vital survival gap was also noticed in the patients with RV failure (83% vs. 63%) [3]. After ten years, follow-up in Algharni et al. reported 90% survival rates in patients with less than moderate tricuspid regurgitation compared to 43% for moderate and severe TR [9]. Individuals with higher grades of TR also had more extended hospital stays and higher renal dysfunction rates and dialysis [18]. They were also more prone to need mechanical circulatory support and required more often redo open chest procedures [3].

Although prompt surgical repair of severe TR that develops early after transplantation is regarded as a safe procedure in selected patients, with an improvement in the overall survival after 1, 5 and 10 years due to better cardiac performance and alleviation of associated organ dysfunction, this redo surgery is not risk-free [11,38]. The postoperative evolution was marked by high rates of prolonged ventilation (33%), new-onset requirement of hemodialysis treatment (36.8%), and infectious complications (11.1%). The reported early mortality was 11.1% [11].

Tricuspid valve annuloplasty had been proven already as a simple, safe, effective, and reliable surgical procedure [39]. Moreover, because it is the least expensive way to treat functional TR, De Vega’s TVA established itself as the treatment of choice for functional TR [39]. The procedure adds little additional time of 5 to 10 min to the operation, the fact that it is also suggested by similar aortic cross-clamp times between the HTX and HTX-A groups [40]. Instead, our results show that TVA contributed to a shorter cardiopulmonary bypass and ischemic time fact attributed to improved right ventricular performance and hemodynamic parameters [23].

TVA has been hypothesized to exert its significant benefits in the early postoperative period [23]. Our meta-analysis of immediate postprocedural, one-week, six-month, and one-year tricuspid insufficiency rates showed significantly lower values in the HTX-A group. This finding would explain the rationale behind establishing the prophylactic donor tricuspid annuloplasty procedure as standard practice. On the other hand, contrary to expected, there was no other significant improvement in the postoperative outcomes. Even though multiple authors have brought strong arguments about the TR’s impact on morbidity and mortality rates, our results revealed no difference in one-year mortality between groups. Unfortunately, the fact that survival data were very heterogenous reported could be why these inexplicable results.

One of the most significant drawbacks of the procedure revolves around the complications involving the conduction system. Rubin and colleagues conducted the most edifying study that focuses on the electrophysiologic consequences associated with tricuspid annuloplasty in heart transplantation. The conduction disturbances reported as significantly more common in the experimental group were the right bundle branch block, left anterior fascicular block, and complete heart block. Permanent pacemaker (PPM) implantation was also more frequent in patients receiving DVA. The authors advise that annuloplasty should be integrated within the context of an equitable tradeoff between the possible risk of conduction abnormalities that occur in the immediate postoperative period and the benefit of preventing late moderate/severe TR [24].

The reported incidence of PPM implantation in heart transplanted patients varies between 5.3% and 10.9% [24,41,42]. Older patients undergoing a biatrial surgical technique with a previous history of amiodarone use are already more susceptible to necessitate pacing without tricuspid intervention [43,44]. Our results showed no difference in the PPM between the groups. However, the negative effect of tissue-damaging during annuloplasty may have been counterbalanced by a shorter ischemic time in the HTX-A group previously reported to contribute to the occurrence of the conduction disturbances [44].

All in one, TA is a simple technique that is worth considering when it comes to orthotopic heart transplantation. The procedure’s aim is clear: to reduce the annulus dilatation development and thus the long-term tricuspid regurgitation. If the results are according to what was initially expected when they were first introduced is still debatable. Correctly performed, it could reduce the risk of severe regurgitation and thus, improve the survival rates and postoperative outcomes while carrying no additional risk for the patient. Some surgeons have discontinued this procedure two years after its implementation, some have assimilated it into the transplantation protocol on the presumption that it has its advantages. However, in the lack of precise data regarding long-term benefits, the basic principle is that TA could be performed as a routine adjunct to orthotopic heart transplantation by experienced surgeons.

### Limitations

This meta-analysis has some significant limitations. First, it includes three observational retrospective studies: a matched case–control study, two prospective nonrandomized studies, and two RCTs. Second, two of the studies authored by the same team of researchers included the same cohort of patients and reported mostly the same outcomes at different periods, the first after a follow-up of 1 year and the second after a follow-up of 5.7 to 6.7 years. Another group of studies authored by the same authors was conducted respecting identical patient selection criteria and the reported outcomes. To avoid biased results, we have considered the meta-analysis of the data presented in the most recent and representative of them. Third, TR was not uniformly graded in all of the studies. Jeevandaman described four degrees of regurgitation, while the authors used a three-stage classification. Fourthly, there were significant discrepancies regarding the surgical technique. Rubin did not report the technique of heart transplantation at all. The patients included in the study conducted by Brown had undergone biatrial heart transplantation, while the other authors used the bicaval technique. TA was performed by De Vega’s technique in all of the studies, except for Brown, who also included the annuloplasties performed using rings.

## 5. Conclusions

Our study showed that donor heart tricuspid annuloplasty reduces tricuspid regurgitation incidence in the first year after orthotopic heart transplantation without increasing the surgical complexity. Further large randomized clinical trials are necessary to evaluate the impact of this procedure on long-term insufficiency and outcome benefits. Regarding one-year and long-term mortality, no significant benefit or harm was revealed. Thus, we emphasize the importance of extending the follow-up period on larger cohorts. In conclusion, if performed in high-experienced centers, prophylactic donor tricuspid annuloplasty could be routinely considered during orthotopic heart transplantation as it tends to incline the balance to a more favorable evolution without adding any additional risks.

## Figures and Tables

**Table 1:** Summary of included studies.

Author	Year	Country	No. of Centers	Type of Study	Time Period	Type of Surgery	Patient Group	No. of Patients Per Group	Follow-Up
Jeevanandam	2004	USA	1	RCT	April 1997–March 1998	Bicaval orthotopic heart transplantation with DeVega TVA	HTX	30	1 year
							HTX-A	30	
Jeevanandam	2006	USA	1	RCT	April 1997–December 2003	Bicaval orthotopic heart transplantation with DeVega TVA	HTX	30	5.7 to 6.7 years
							HTX-A	30	
Rubin, G	2018	USA	1	Retrospective observational	2013–2017	Orthotopic heart transplantation with DeVega TVA	HTX	104	32 months
							HTX-A	76	
Greenberg J	2017	USA	18	Retrospective observational- Propensity score-matched	January 2002–December 2016	Bicaval orthotopic heart transplantation with DeVega TVA	HTX	117	7.9 ± 4.3 years
							HTX-A	130	5.2 ± 2.9 years
Fiorelli	2007	Brazil	1	Prospective Observational- nonrandomized	March 1985–December 2005	Bicaval orthotopic heart transplantation with DeVega TVA	HTX	10	14.6 ± 4.3 months
							HTX-A	10	
Fiorelli	2010	Brazil	1	Prospective Observational- nonrandomized	2002–2010	Bicaval orthotopic heart transplantation with DeVega TVA	HTX	15	26.9 ± 5.4 months
							HTX-A	15	
Brown	2004	USA	1	Retrospective Observational	November 1999–July 2001	Biatrial cardiac transplantation with a Cabrol modification with either a DeVega (n = 10) or Ring (n = 15) TVA	HTX	25	6 months
							HTX-A	25	

HTX—heart transplantation; HTX-A—heart transplantation with tricuspid annuloplasty; TVA—tricuspid valve annuloplasty.

**Table 2:** Baseline characteristics and periprocedural data.

Parameters	No. of Studies	No. of HTX Patients	No. of HTX-A Patients	HTX Mean ± SD or (%)	HTX-A Mean ± SD or (%)	*p*-Value
Demographics						
Age	5	331	319	51.48 ± 10.20	51.92± 11.32	0.6
Male	5	331	319	72.2%	73.3%	0.8
Preoperative data						
Ischemic etiology of the end-stage heart failure	4	132	148	88.63%	67.57%	0.0001
Inotropic medication	2	172	188	30.23%	37.76%	0.2
Preoperative renal function						
Creatinine	2	187	203	1.26 ± 0.93	1.22 ± 0.46	0.6
BUN	2	187	203	23.73 ± 11.90	23.56 ± 11.82	0.9
Hemodynamic parameters						
Pulmonary capillary wedge pressure	2	187	203	17.16 ± 8.55	19.71± 9.14	0.005
Pulmonary vascular resistance woods units	5	298	300	2.29 ± 1.00	2.17 ± 1.02	0.15
Mechanical circulatory support	4	306	304	55.88%	52.63%	0.5
Intraoperative times						
CPB duration	3	144	116	173.33± 27.75	154.15 ± 25.89	<0.0001
Ischemic time	5	326	314	181.75 ± 40.83	165.32 ± 41.72	<0.0001
Aortic cross-clamp	4	169	141	88.02 ± 20.50	86.89± 13.86	0.6

## Data Availability

The data presented in this study are available on request from the corresponding author.

## Supplementary Materials

The following are available online at https://www.mdpi.com/2227-9032/9/3/306/s1, Table S1: preferred reporting items for systematic reviews and meta-analysis (PRISMA) checklist, Table S2: Risk of publication bias assessment.
